# Serum SP-A and KL-6 levels can predict the improvement and deterioration of patients with interstitial pneumonia with autoimmune features

**DOI:** 10.1186/s12890-020-01336-y

**Published:** 2020-12-02

**Authors:** Jingxian Wang, Peiyan Zheng, Zhifeng Huang, Huimin Huang, Mingshan Xue, Chenxi Liao, Baoqing Sun, Nanshan Zhong

**Affiliations:** 1 Department of Allergy and Clinical Immunology, The First Affiliated Hospital of Guangzhou Medical University, Guangzhou Medical University, 151 Yanjiang West Road, Guangzhou, 510120 China; 2 National joint local engineering laboratory for Cell Engineering and Biomedicine Technique, Gui zhou Province Key Laboratory of Regenerative Medicine, Key Laboratory of Adult Stem Cell Translational Research (Chinese Academy of Medical Sciences), Guizhou Medical University, Guiyang, China

**Keywords:** Interstitial pneumonia with autoimmune features, Non-fibrotic lung diseases, Surfactant protein-a, Krebs von den Lungen-6

## Abstract

**Background:**

Some patients with interstitial pneumonia with autoimmune features (IPAF) showed a progressive course despite therapy. This study aimed to evaluate whether serial changes in the serum levels of surfactant protein-A (SP-A) and Krebs von den Lungen-6 (KL-6) can predict disease progression.

**Methods:**

Sixty-four patients with IPAF and 41 patients with non-fibrotic lung disease (non-FLD) were examined. Based on long-term changes in lung function, 36 IPAF patients who were followed up for more than 3 months were divided into a progressive group (*n* = 9), an improvement group (*n* = 13), and a stable group (*n* = 14). Serum KL-6 and SP-A levels were measured. The sensitivity, specificity, cut-off value, and area under the curve (AUC) value for each of the indices were determined using receiver operating characteristic (ROC) curve analysis. The expression differences in these biomarkers and their correlation with disease severity were analyzed.

**Results:**

Compared with non-FLD patients, serum SP-A and KL-6 levels in IPAF patients were increased significantly [SP-A: (*p* < 0.001); KL-6: (*p* < 0.001)] and negatively correlated with DLCO (SP-A: r_S_ = − 0.323, *p* = 0.018; KL-6: r_S_ = − 0.348, *p* = 0.0011). In patients with progressive disease, the posttreatment serum SP-A and KL-6 levels were increased significantly compared with pretreatment levels [SP-A: (*p* = 0.021); KL-6: (*p* = 0.008)]. In patients showing improvement, the levels were decreased significantly [SP-A (*p* = 0.007) and KL-6 (*p* = 0.002)]. Changes in serum biomarkers (Delta SP-A and Delta KL-6) were significantly negatively correlated with changes in lung function (Delta FVC, Delta DLCO and Delta FEV1) (r_S_ = 0.482, *p* < 0.05). A significant positive correlation was found between Delta SP-A and Delta KL-6 (r_S_ = 0.482, *p* < 0.001).

**Conclusions:**

Serum SP-A and KL-6 offer high sensitivity and specificity for the diagnosis of IPAF. The decrease in serum SP-A and/or KL-6 levels in patients with IPAF is related to the improvement in pulmonary function. SP-A and KL-6 may be important biomarkers for predicting disease progression in patients with IPAF.

## Background

Interstitial pneumonia with autoimmune features (IPAF) is a new term that was proposed by the joint research statement of the European Respiratory Society and American Thoracic Society (ERS/ATS) [1]. IPAF refers specifically to idiopathic interstitial pneumonia (IIP). IPAF shares some characteristics with connective tissue disease (CTD), but it cannot be diagnosed as a certain type of CTD. IPAF is clinically located in the cross-domain of IIP and CTD-interstitial lung disease (CTD-ILD). At present, the diagnosis of IPAF is based mainly on clinical manifestations, pulmonary imaging and lung histopathology [2]. ILD is the main manifestation among patients, and there are some serological autoantibody-positive or multisystem extrapulmonary manifestations, such as morning stiffness, Raynaud’s phenomenon, and dry symptoms [3]. Lung function tests are extremely sensitive to the early changes in IPAF, with a sensitivity that is even higher than that of high-resolution computed tomography (HRCT) [2]. Changes in lung function can also reflect the progression of IPAF and determine the effect of treatment and prognosis. However, lung function tests are not suitable for critically ill patients. Therefore, the identification of a more convenient, reliable, and accurate diagnostic method is of great significance for the screening, treatment, and dynamic evaluation of IPAF. The identification of biomarkers for IPAF will not only improve the level of IPAF diagnosis but will also aid in the understanding of the pathophysiological mechanism of the occurrence and development of IPAF. To date, there are few studies on IPAF biomarkers.

Surfactant protein-A (SP-A) and Krebs von den Lungen-6 (KL-6) are proteins expressed in type II alveolar epithelial cells and are related to the pathogenesis of pulmonary fibrosis [4, 5]. The expression level of KL-6 significantly increases in alveolar tissue affected by interstitial pneumonia and enters the blood circulation through the damaged alveoli [6]. The alveolar surface protein SP-A, which is synthesized and secreted by airway and alveolar epithelial cells, is an important marker of alveolar injury [7, 8]. In Japan [9, 10], serum KL-6, SP-A and SP-D levels are widely used as biomarkers for the diagnosis and prognosis of idiopathic pulmonary fibrosis and ILD. While KL-6 has high specificity and sensitivity in the diagnosis of interstitial lung diseases, SP-A can well distinguish idiopathic pulmonary fibrosis (IPF) from other ILDs [11]. In IPF, the serum levels of KL-6 and SP-A are associated with disease severity at the time of measurement and with long-term outcomes [12, 13]. However, few studies have examined the correlation between these biomarkers in IPAF and disease severity.

Previous clinical trials or observational studies on patients with ILD have usually defined ILD progression as a decline in forced vital capacity (FVC), typically by 10% of the predicted value [14]. Lee et al. [15] defined disease improvement or ILD progression as changes in FVC ≥10% and/or changes in diffusing capacity for carbon monoxide (DLCO) ≥15%. Jiang et al. [16] defined progression as mortality or a reduction in FVC by > 10% and/or DLCO by > 15%. According to the above mentioned criteria, 36 patients with IPAF who were followed up for > 3 months were divided into three groups: progressive group (9 patients), improvement group (13 patients) and stable group (14 patients).

In the present study, we determined the serum SP-A and KL-6 levels in patients with IPAF, analyzed the correlation between their expression levels and lung function indicators, and explored further changes in the above mentioned marker levels during disease progression, providing guidance for early diagnosis and condition monitoring.

## Methods

### Study design

This study included two parts. The purpose of the first part was to compare the serum SP-A and KL-6 levels between patients diagnosed with IPAF and those diagnosed with non-fibrotic lung disease (non-FLD) and study their diagnostic value. The purpose of the second part was to compare serum SP-A and KL-6 levels before and after treatment and evaluate their prognostic value. The research scheme was approved by the Institutional Ethics Committee of the First Affiliated Hospital of Guangzhou Medical University (ethics approval no. Gyfyy-2016-73).

### Diagnostic criteria and treatment

We retrospectively investigated 64 patients with IPAF diagnosed at the First Affiliated Hospital of Guangzhou Medical University between October 2015 and February 2019 according to the diagnostic criteria for IPAF established by the ERS/ATS in 2015. These classification criteria are based on a combination of features from three domains: a clinical domain consisting of extra-thoracic features; a serologic domain with specific autoantibodies; and a morphologic domain with imaging patterns, histopathological findings or multi-compartment involvement. IPAF was confirmed when the patients showed the clinical and/or serological domain criteria specified by the ERS/ATS task force [1].

Sixty-four IPAF patients were initially enrolled: 13 patients (20.3%) met the clinical manifestations and serological manifestations, 16 patients (25%) met the clinical manifestations and morphological manifestations, and 35 patients (54.7%) met the serological manifestations and morphological manifestations. A total of 10 patients (15.6%) met all three criteria.

At present, there is no expert consensus or guidelines on the treatment of IPAF. The treatment approach comes mainly from the approach for connective tissue disease-related interstitial lung disease (i.e., glucocorticoids alone or in combination with azathioprine, cyclophosphamide, pirfenidone and so on).

Among the 64 IPAF patients, 35 were treated with prednisone, 21 were treated with prednisone plus cyclophosphamide, 1 was treated with prednisone plus cyclophosphamide and pirfenidone, 2 were treated with pirfenidone plus cyclophosphamide, 2 were treated with prednisone plus pirfenidone, and 3 were treated with pirfenidone.

Pregnant women, patients with malignant tumours or other autoimmune diseases or co-infections, and patients aged < 18 years were excluded from the study.

Forty-one patients with non-FLD were used as disease controls. Of these 41 subjects, 13 had chronic obstructive pulmonary disease, 10 had lung cancer, 10 had bacterial pneumonia, 2 had eosinophilic pneumonia, 1 had bronchiectasis, 1 had chronic bronchitis, 1 had emphysema, 1 had asthma, 1 had granuloma, and 1 had pulmonary tuberculosis. All diseases met their diagnostic criteria.

The 36 patients with IPAF who received treatment were followed up for > 3 months. The following data were collected from the patients’ medical records: gender, age, body mass index (BMI), smoking history, and lung function.

### Lung function measurements

According to the recommendations of the ERS/ATS, lung function tests were performed on a computerized spirometer (MasterScreen, Leibnizstrasse, Hoechberg, Germany). The examination parameters included FVC, forced expiratory volume in 1 s (FEV1), and DLCO.

### Blood collection

In the 64 patients with IPAF, the initial symptoms included shortness of breath (41/64, 64.1%), cough (37/64, 57.8%), expectoration (26/64, 40.6%), chest pain and chest tightness (15/64, 23.4%), Dyspnea occurred (5/64, 7.8%) and fever (3/64, 4.7%). Meanwhile, the concomitant symptoms and signs exhibited in some of the patients included inflammatory arthritis and polyarticular morning joint stiffness (8/64, 12.5%), Raynaud phenomenon (2/64, 3.1%), finger swelling (2/64, 3.1%), dry mouth and dry eyes (2/64, 3.1%), muscle soreness (2/64, 3.1%), edema of both lower limbs (1/64, 1.6%), and palpitation (1/64, 1.6%). The symptoms in each patient persisted during the course of the disease.

The fasting morning blood (5 mL) of the patients were collected within 24 h of the onset of the first respiratory symptoms via coagulation-promoting tubes. The collected samples were stood for about 30 min at room temperature and centrifuged at 3000 r/min for 10 min to obtain serum. Aliquots of serum were stored at − 80 °C to avoid repeated freezing and thawing.

### Measurement of serum SP-A and KL-6 levels

Serum SP-A and KL-6 levels were measured on a fully automatic immunoanalyser, HISCL-5000 (Sysmex Corp., Hyogo, Japan), according to the manufacturer’s instructions. The detection range for the SP-A level was 1–1000 ng/mL and that for KL-6 was 10–6000 U/mL. Samples that were above the upper detection limit were excluded from the analysis. SP-A and KL-6 assay kits were obtained from Sysmex Corporation.

### Definitions of disease progression, improvement, and stable condition

Disease progression was defined as a decrease in FVC ≥10% and/or DLCO ≥15%. Disease improvement was defined as an increase in FVC by ≥10% and/or DLCO by ≥15%. Stable condition was defined as a change in FVC by < 10% and DLCO by < 15%.

### Statistical analysis

The normality of continuous variables was assessed with the Shapiro-Wilk test, and the data are expressed as the mean ± standard deviation or median plus interquartile range (25–75th percentiles) according to their distribution (normal or non-normal). Dichotomous data are presented as frequencies and percentages. The chi-squared test or Fisher’s exact test was used to analyse the differences in categorical data. Differences in the levels of the various serum markers between subject groups were analysed using the Kruskal-Wallis H test and Wilcoxon’s rank-sum test. Correlation analyses were performed using Spearman’s rank correlation. A receiver operating characteristic (ROC) curve was prepared to analyse the specificity and sensitivity for SP-A and KL-6 for disease activity. All statistical analyses were performed using the SPSS statistical software package for Windows (version 22.0; SPSS Inc., Chicago, IL, USA). *P* values < 0.05 were considered significant.

## Results

### Clinical data of subjects

This study included 64 IPAF patients (35 females and 29 males), with an average age of 51.5 ± 13.15 years and an average BMI of 24.33 ± 3.29 kg/m^2^. Eighteen (28.13%) were smokers. This study also included 41 non-FLD patients (14 females and 27 males), with an average age of 54.7 ± 11.55 years and an average BMI of 25.49 ± 3.46 kg/m^2^. Twelve (29.27%) were smokers (Table 1). The results revealed no significant differences in age, gender, BMI, or smoking history between patients with IPAF and those with non-FLD.

**Table 1:** Baseline characteristics in patients with IPAF and Non-FLD

	IPAF	Non-FLD	***P*** value
**Number, n**	64	41	–
**Age, year**	51.5 ± 13.15	54.7 ± 11.55	0.979
**Female, n (%)**	35 (54.69%)	24 (58.54%)	0.698
**BMI (kg/m2)**	24.33 ± 3.29	25.49 ± 3.46	0.194
**Smoker, n (%)**	18 (28.13%)	12 (29.27%)	0.899
**DLCO (%Pred)**	55.05 ± 12.9	–	–
**FVC (%Pred)**	70.11 ± 17.75	–	–
**FEV1 (%Pred)**	73.15 ± 16.6	–	–

The data are presented as means ± standard deviation. Other variables are presented as numbers (percent). *IPAF* Interstitial pneumonia with autoimmune features, *Non-FLD* non-fibrotic lung diseases, *BMI* body mass index, *DLCO* diffusing capacity for carbon monoxide, *FVC* forced vital capacity, *FEV1* forced expiratory volume in 1 s; %Pred, percent predicted

### Comparison of serum KL-6 and SP-A levels between non-FLD patients and IPAF patients

**Fig. 1:**
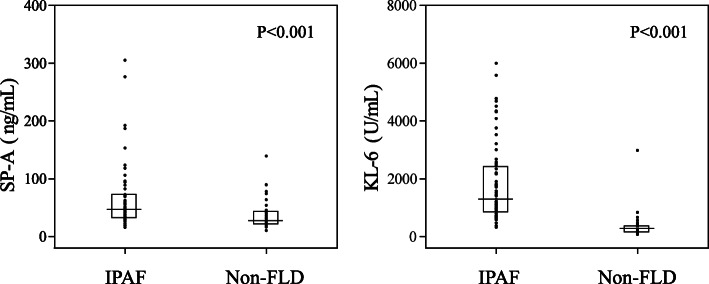
Comparison of serum SP-A and KL-6 levels in Non-FLD and IPAF patients. IPAF, Interstitial pneumonia with autoimmune features; Non-FLD, Non-fibrotic lung diseases; SP-A, Surfactant protein-A; KL-6, Krebs von den Lungen-6. The data was presented as median with interquartile range

**Fig. 2:**
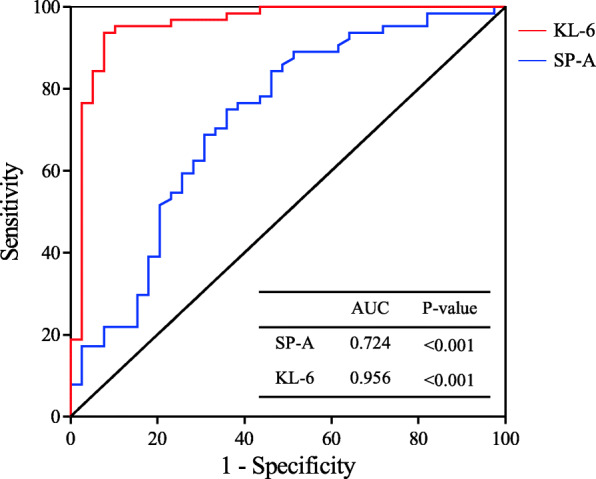
Receiver-operating characteristic (ROC) curve according to the specificity and sensitivity of serum SP-A and KL-6 levels. SP-A, surfactant protein-A; KL-6, Krebs von den Lungen-6

The serum SP-A level in IPAF patients was 46.6 (32.38–72.58) ng/mL, which was significantly higher than that in non-FLD patients (22.3 (27.7–43.7) ng/mL) (*p* < 0.001). Similarly, the serum KL-6 level in IPAF patients was 1315.5 (848.75–2416.75) U/mL, which was significantly higher than that in non-FLD patients (299 (152–369) U/mL) (*p* < 0.001) (Fig. 1). We also used ROC curve analysis to evaluate the sensitivity and specificity of serum SP-A and KL-6 concentrations as biomarkers for the diagnosis of IPAF (Fig. 2). Based on the area under the ROC curve, when the cut-off level for SP-A to distinguish IPAF was 32.75 ng/mL, the sensitivity and specificity were 75 and 64.2%, respectively (AUC = 0.724, 95% CI = 0.619–0.829). When the cut-off level for KL-6 to distinguish IPAF was 562.5 U/mL, the sensitivity and specificity were 93.8 and 92.3%, respectively (AUC = 0.956, 95% CI = 0.911–1.000).

### Correlations between biomarkers and pulmonary function

**Fig. 3:**
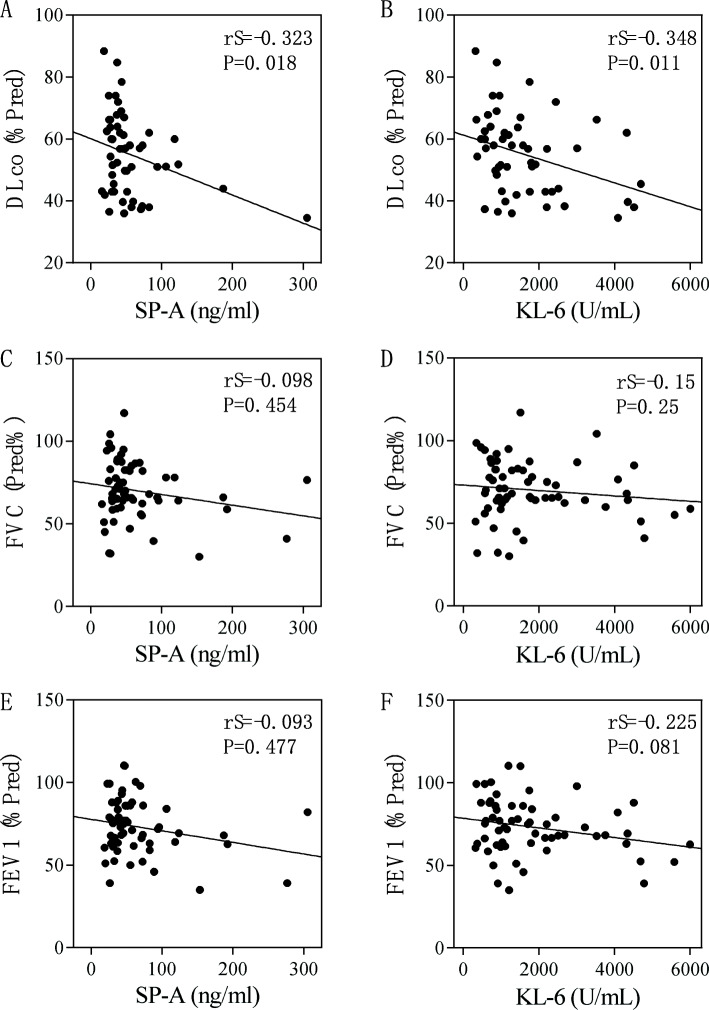
Correlation between serum SP-A and KL-6 concentrations and pulmonary function test parameters in IPAF patients using Spearman correlation test. SP-A, surfactant protein-A; KL-6, Krebs von den Lungen-6; DLCO, diffusing capacity for carbon monoxide; FVC, forced vital capacity; FEV1, forced expiratory volume in 1 s

Both biomarkers (SP-A and KL-6) showed significant negative correlations with DLCO (%Pred) (SP-A: rS = − 0.323, *p* = 0.018; KL-6: rS = − 0.348, *p* = 0.0011) (Fig. 3a, b). However, there was no significant correlation between SP-A and KL-6 levels and FVC (%Pred) (SP-A: rS = − 0.098, *p* = 0.454; KL-6: rS = − 0.15, *p* = 0.25) (Fig. 3c, d). Similarly, SP-A and KL-6 did not show a significant correlation with FEV1 (%Pred) (SP-A: rS = − 0.093, *p* = 0.477; KL-6: rS = − 0.225, *p* = 0.081) (Fig. 3e, f).

### Analysis of serum SP-A and KL-6 levels before and after treatment

**Fig. 4:**
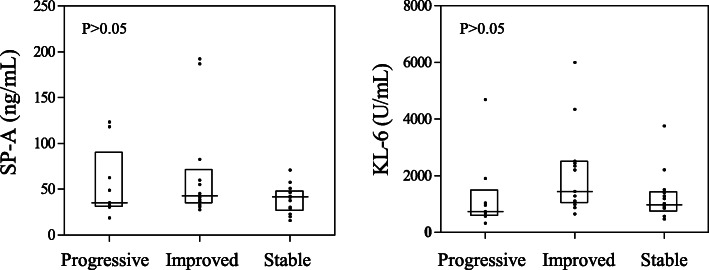
Comparison of serum SP-A and KL-6 levels in the progress, improve and stable groups before treatment. SP-A, surfactant protein-A; KL-6, Krebs von den Lungen-6. The data was presented as median with interquartile range

**Fig. 5:**
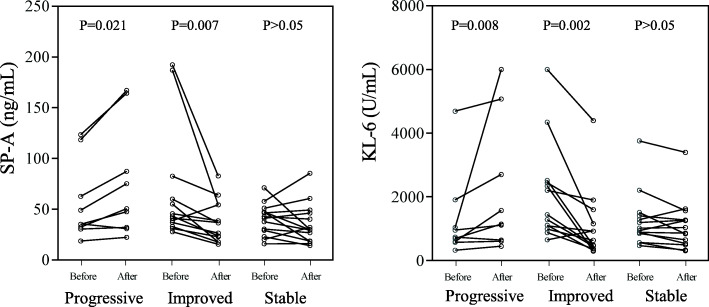
Pretreatment and posttreatment serum SP-A and KL-6 levels compared between the three groups. SP-A, surfactant protein-A; KL-6, Krebs von den Lungen-6

To determine the value of serum SP-A and KL-6 levels in the evaluation of therapeutic efficacy in patients with IPAF, patients with IPAF who were followed up for > 3 months were divided into a progressive group (*n* = 9), an improvement group (*n* = 13) and a stable group (*n* = 14) according to changes in pulmonary function. The patients’ clinical baseline characteristics were shown in Table 2. There were no significant differences in any of the parameters between the three groups. The Kruskal-Wallis H test was used to compare the serum SP-A and KL-6 levels of the three groups of patients before treatment, and the results were not significantly different (*p* > 0.05) (Fig. 4). Subsequently, we compared the levels of SP-A and KL-6 in patients with IPAF before and after treatment by Wilcoxon′s rank-sum test (Fig. 5). In the progressive group, the levels of serum SP-A [35.3 (31.35–90.4) ng/mL versus 50.3 (31.35–125.75) ng/mL (*p* = 0.021)] and KL-6 [738 (584–1471) U/mL versus 1143 (676.5–3888) U/mL (*p* = 0.008)] were increased significantly after treatment. Compared with before treatment, the levels of serum SP-A [42.9 (34.85–71.2) ng/mL versus 36.5 (20.25–54.25) ng/mL (*p* = 0.007)] and KL-6 [1440 (1039.5–2478) U/mL versus 635 (407–1379.5) U/mL (*p* = 0.002)] in the improvement group were decreased significantly after treatment. In the stable group, serum SP-A [41.75 (27.475–48.125) ng/mL versus 29.65 (18.6–46.95) ng/mL (*p* > 0.05)] and KL-6 [979.5 (777.75–1430.25) U/mL versus 949 (523.5–1347.25) U/mL (*p* > 0.05)] levels did not change significantly compared with those before treatment.

**Table 2:** Baseline characteristics of the three groups of patients with IPAF

	Progressive	Improved	Stable	***P*** value
**Number, n**	9	13	14	–
**Age, year**	53.78 ± 17.18	53.38 ± 10.85	53.44 ± 12.16	0.996
**Female, n (%)**	5 (55.55%)	9 (69.23%)	8 (57.14%)	0.753
**BMI (kg/m**^**2**^**)**	24.36 ± 3.05	24.13 ± 4.49	24.38 ± 2.19	0.979
**Smoker, n (%)**	2 (22.22%)	2 (15.38%)	5 (35.71%)	0.379
**DLCO (% Pred)**	62.37 ± 14.43	54.05 ± 12.8	53.85 ± 13.54	0.355
**FVC (% Pred)**	68.59 ± 11.92	69.76 ± 10.82	76.25 ± 19.82	0.220
**FEV1 (% Pred)**	73.74 ± 14.49	70.44 ± 10.85	80.6 ± 18	0.422

*IPAF* Interstitial pneumonia with autoimmune features, *BMI* body mass index, *DLCO* diffusing capacity for carbon monoxide, *FVC* forced vital capacity, *FEV1* forced expiratory volume in 1 s, *SP-A* surfactant protein-A, *KL-6* Krebs von den Lungen-6, *%Pred* percent predicted. The data are presented as median with interquartile range or as number (percentage)

### Correlations between changes in Delta KL-6 and Delta SP-A and changes in pulmonary function

**Fig. 6:**
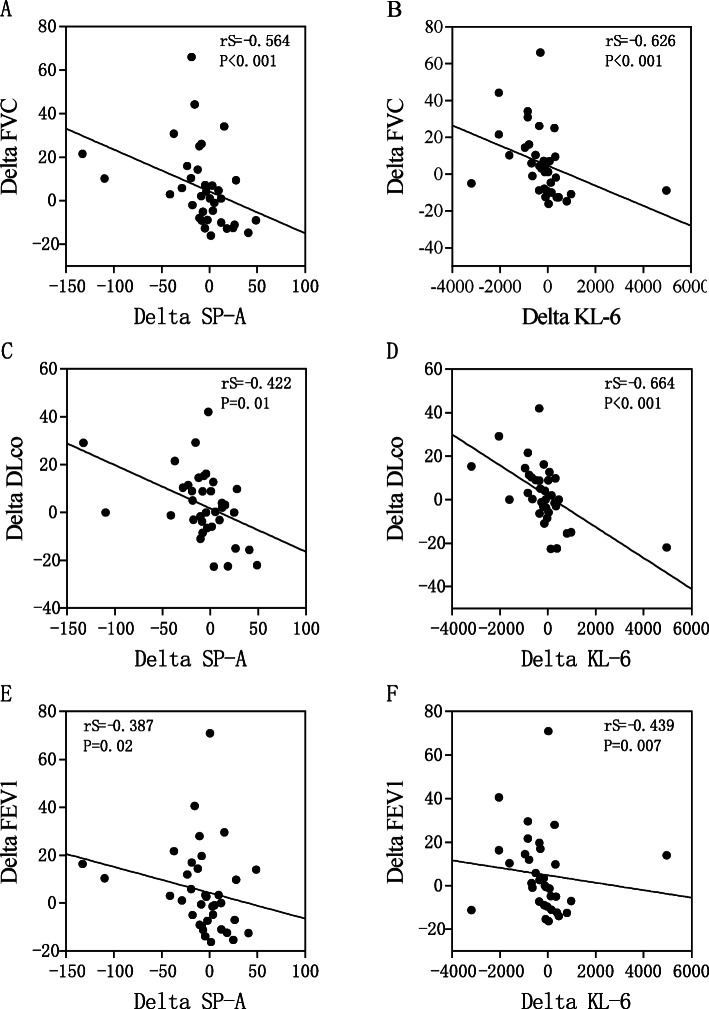
Correlations between Delta SP-A and Delta KL-6 and changes in pulmonary function test parameters. SP-A, surfactant protein-A; KL-6, Krebs von den Lungen-6; DLCO, diffusing capacity for carbon monoxide; FVC, forced vital capacity; FEV1, forced expiratory volume in 1 s

**Fig. 7:**
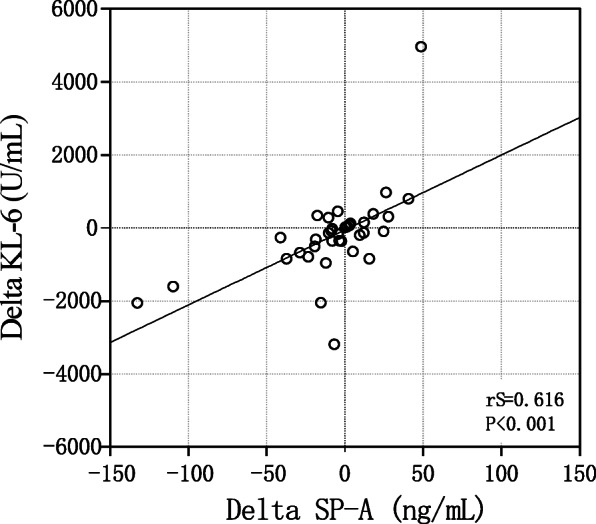
Correlation between serum Delta KL-6 and Delta SP-A values. SP-A, surfactant protein-A; KL-6, Krebs von den Lungen-6

We also used Spearman′s correlation test to study the correlation between changes in serum biomarkers (Delta SP-A and Delta KL-6) and changes in lung function (Delta FVC, Delta DLCO and Delta FEV1) before and after treatment. The serum levels of Delta SP-A showed a significant inverse correlation with Delta FVC, Delta DLCO and Delta FEV1 (FVC: r_S_ = − 0.564, *p* < 0.001; DLCO: r_S_ = − 0.422, *p* = 0.01; FEV1: r_S_ = − 0.387, *p* = 0.02) (Fig. 6a, c and e). Delta KL-6 also showed a significant inverse correlation with Delta FVC, Delta DLCO and Delta FEV1 (FVC: r_S_ = − 0.626, *p* < 0.001; DLCO: r_S_ = − 0.664, *p* < 0.001; FEV1: r_S_ = − 0.439, *p* = 0.007) (Fig. 6b, d and f). We also found a significant positive correlation between Delta SP-A and Delta KL-6(r_S_ = 0.616, *p* < 0.001; Fig. 7).

## Discussion

KL-6 is a MUC-1 mucin, that is commonly found in regenerative type II alveolar epithelial cells [17, 18]. Interstitial pneumonia will promote the proliferation of type II alveolar epithelial cells, resulting in an increase in the KL-6 concentration, and this damage leads to an increase in vascular permeability, allowing KL-6 to enter the bloodstream; therefore, the concentration of KL-6 in the serum of patients with ILD increases [19, 20]. SP-A is a member of the water-soluble C-type lectin family and is an important part of the lung’s innate immune system [21]. The pathogenesis of IPF may be related to the abnormal endoplasmic reticulum processing of lung surfactant proteins [22]. Based on the genetic analysis of lung biopsy samples from IPF patients, the expression of the SP-A1 gene is upregulated, and SP-A2 gene defects are associated with the pathogenesis of familial IPF [23, 24]. In Japan, serum SP-A and KL-6 levels are widely used as biomarkers for the diagnosis, severity assessment and prognosis prediction of ILD patients [9]. These findings collectively indicate that serum SP-A and KL-6 can act as a surrogate markers for the active process of disease progression [25, 26]. However, it is not known whether changes in SP-A and KL-6 levels, especially in the serum of patients with IPAF, can reflect the correlation between the changes in and the progression of IPAF patients.

Our study found that compared with the non-FLD group, the serum levels of SP-A and KL-6 were significantly increased in IPAF patients (*p* < 0.01). These results show that serum KL-6 and SP-A can well distinguish IPAF patients from non-FLD patients. Our findings are consistent with the results from a report by Xue et al. [27]. When a cut-off value of 32.75 ng/mL was used, the sensitivity and specificity of using the serum SP-A level as a diagnostic biomarker were 75 and 64.2%, respectively. When a cut-off value of 562.5 U/mL was used, the sensitivity and specificity of using the serum KL-6 level as a diagnostic biomarker were 93.8 and 92.3%, respectively. These findings suggest that in contrast to non-FLD, serum KL-6 may be a promising biomarker for the diagnosis of IPAF. In IPF patients, the following cut-off values were set as the levels that resulted in the optimal diagnostic accuracy for SP-A and KL-6: 476 U/mL for KL-6 and 44.0 ng/mL for SP-A [9]. These results suggest that different levels of criticality may be required for ILD patients with different subtypes.

In IPAF, serum SP-A and KL-6 levels were significantly negatively correlated with %DLCO (*p* < 0.05), but no significant correlation with %FVC or %FEV1 (*p* > 0.05), respectively. Most IPAF patients were diagnosed as mixed ventilation dysfunction and pulmonary diffusion dysfunction in the pulmonary function test (PFT). Diffusion function is a sensitive index for early diagnosis. Ahmad K et al. considered that diffusion dysfunction played an important role in clinical diagnosis, because it can be the primary manifestation of interstitial pneumonia, and its sensitivity was found to be higher than the change of lung volume [28]. Therefore, compared with %FVC, %DLCO may reflect the severity of IPAF more accurately before treatment. Despite of that, the measurement of %DLCO could be more difficult, especially for patients with more severe symptoms because it requires cooperation of patients. Hence, it is necessary to analyzed the changes in both %DLCO and %FVC.

Among the patients who received follow-up, the levels of serum SP-A and KL-6 in those with progressive disease were significantly higher after treatment than before treatment, while the levels of serum SP-A and KL-6 were significantly lower in those with improved conditions, suggesting that serum SP-A and KL-6 may be effective biomarkers for monitoring the progression of IPAF. Arai et al. [24] reported that the periodic measurement of KL-6 and SP-D levels would be useful in the evaluation of disease progression and treatment response in patients with idiopathic fibrotic nonspecific interstitial pneumonia.

We used Spearman’s correlation test to study the correlation between Delta SP-A, Delta KL-6 and changes in lung function parameters (Delta DLCO, Delta FVC and Delta FEV1) to further explore the roles of SP-A and KL-6 in the monitoring of prognoses in patients with IPAF. Delta SP-A and Delta KL-6 were significantly negatively correlated with Delta DLCO, Delta FVC, and Delta FEV1 (*P* < 0.01). The initial KL-6 and SP-A levels correlated inversely with %DLCO at the time of IPAF diagnosis. In CTD-associated interstitial pneumonia, the serum levels of KL-6 and SP-D are negatively correlated with FVC and %DLCO [24, 28–31]. In the report by Lee et al. [15], serum SP-A and KL-6 levels in CTD-ILD patients were significantly negatively correlated with FVC and DLCO. This study reported similar negative correlations between KL-6 and respiratory parameters [32]. This study also confirmed the relationship between SP-A and KL-6 with disease activity, suggesting that SP-A can also be used as an indicator to predict the prognosis of IPAF. Therefore, we confirmed that the serum levels of SP-A and KL-6 reflect the severity of IPAF in terms of pulmonary function deterioration.

The diagnosis of IPAF is based on the results of HRCT or invasive transbronchial lung biopsy, which can be influenced by numerous factors or bring great suffering to patients [33]. Although lung function tests are non-invasive, they are highly dependent on the cooperation of the patient. Furthermore, respiratory failure due to the acute exacerbation of ILD often inhibits patients from properly performing PFT [34]. Compared with frequent PFT, X-ray examinations and invasive bronchial lung biopsy, it is easier to detect serum KL-6 and SP-A during the entire disease course [33]. The relevant analysis performed in this study showed that when lung function tests are difficult, we can make a preliminary assessment and prediction of the patient’s condition based on the expression levels of the above two markers.

Our results also showed that although there was a significant correlation between Delta SP-A and Delta KL-6, the correlation coefficient was not high, suggesting that each marker may represent a different pathophysiological mechanism.

Our study had some limitations. First of all, due to the retrospective nature of the study, data related to symptom initiation and partial examination were missing. There was no analysis of HRCT manifestations or 6-min walking experiments. Second, the sample size of this study was small. Future studies with larger sample sizes are needed to verify our findings. Third, a retrospective analysis of the disease course does not allow changes in treatment and follow-up time, so it is difficult to avoid the impact of other confounding factors. A prospective study with a larger sample size is needed to examine the effect of KL-6 and SP-A on other prognostic parameters of patients with IPAF, such as HRCT findings and dyspnoea score.

In summary, this study showed that the levels of serum SP-A and KL-6 in patients with IPAF were significantly higher than those in patients with non-FLD and negatively correlated with %DLCO. The levels of SP-A and KL-6 increased with disease progression and decreased with disease remission. To the best of our knowledge, we are the first to report changes in serum SP-A and KL-6 levels with disease progression in patients with IPAF.

## Conclusions

In conclusion, serum SP-A and KL-6 levels were significantly higher in patients with IPAF than in patients with non-FLD. The serum KL-6 and SP-A levels of IPAF patients in the improved group were significantly decreased, while they were significantly increased in the progressive group. The levels of serum SP-A and KL-6 reflect the severity of pulmonary function deterioration in IPAF. We believe that regular measurements of KL-6 and SP-A levels can be used as a strategy for the diagnosis and assessment of disease progression.

## Data Availability

The datasets generated and/or analyzed during the current study are not publicly available but are available from the corresponding author on reasonable request.

## Abbreviations

IPAF: Interstitial pneumonia with autoimmune features
SP-A: Surfactant protein-A
KL-6: Krebs von den Lungen-6
AUC: Area under the curve
ROC: Receiver operating characteristic
Non-FLD: Non-fibrotic lung diseases
ERS/ATS: European Respiratory Society and American Thoracic Society
IIP: Idiopathic interstitial pneumonia
CTD: Connective tissue disease
CTD-ILD: Connective Tissue Disease-Interstitial lung disease
HRCT: High-resolution computed tomography
FVC: Forced vital capacity
DLCO: Diffusing capacity for carbon monoxide
FEV1: Forced expiratory volume of 1 s
BMI: Body mass index
